# Lower incidence of myocardial infarction after smoke-free legislation enforcement in Chile

**DOI:** 10.2471/BLT.16.189894

**Published:** 2017-08-28

**Authors:** Carolina Nazzal, Jeffrey E Harris

**Affiliations:** a School of Public Health, Faculty of Medicine, University of Chile, Santiago, Chile.; b Department of Economics, Massachusetts Institute of Technology, 77 Massachusetts Avenue, Cambridge, Massachusetts, MA 02139, United States of America.

## Abstract

**Objective:**

To evaluate the impact of a complete smoking ban in enclosed spaces on the incidence of acute myocardial infarction in Chile.

**Methods:**

The population-based study involved residents of urban areas, where 80% of the Chilean population live, aged 20 years or older who had a myocardial infarction. Monthly myocardial infarction incidence and mortality rates at health-care facilities between January 2011 and December 2014 were derived from admission and mortality databases. Regression discontinuity methods were used to estimate the near-immediate impact on disease incidence of enforcing smoke-free legislation in March 2013. The same analysis was performed for ischaemic stroke, degenerative disc disease and colon cancer. Data on the concentration of fine respirable particulates were included in an additional analysis of myocardial infarction incidence in the Santiago metropolitan area.

**Results:**

The enforcement of smoke-free legislation was associated with an abrupt, near-immediate decline of 0.639 cases of myocardial infarction per 100 000 adults per month (95% confidence interval, CI: 0.242 to 1.036; relative decline: 7.8%). Similar declines were observed in men and women and in people aged over and under 70 years. However, enforcement of the legislation was not associated with a significant change in the rate of ischaemic stroke, degenerative disc disease or colon cancer. The abrupt decline in myocardial infarction incidence was also observed when data on fine respirable particulates were included in an analysis for Santiago.

**Conclusion:**

The enforcement of extensive smoke-free legislation in Chile was associated with an abrupt, near-immediate decline in the incidence of myocardial infarction.

## Résumé

**Objectif:**

Évaluer l'impact d'une interdiction totale de fumer dans les espaces fermés sur l'incidence d'infarctus aigus du myocarde au Chili.

**Méthodes:**

Une étude a été menée auprès des habitants des zones urbaines – où se concentre 80% de la population chilienne – âgés de 20 ans ou plus et victimes d'un infarctus du myocarde. Les taux mensuels d'incidence d'infarctus du myocarde et de mortalité au sein des établissements de santé entre janvier 2011 et décembre 2014 ont été calculés à partir de bases de données sur les admissions et la mortalité. Des méthodes de régression sur discontinuité ont été utilisées pour estimer l'impact quasi immédiat de l'entrée en vigueur, en mars 2013, de la législation antitabac sur l'incidence de la maladie. La même analyse a été réalisée à l'égard des AVC ischémiques, de la discopathie dégénérative et du cancer du côlon. Des données relatives à la concentration de particules fines respirables ont été incluses dans une analyse complémentaire de l'incidence d'infarctus du myocarde dans la région métropolitaine de Santiago.

**Résultats:**

L'entrée en vigueur de la législation antitabac a été associée à un déclin brusque, quasi-immédiat, de 0,639 cas d'infarctus du myocarde pour 100 000 adultes par mois (intervalle de confiance, IC, à 95 %: de 0,242 à 1,036; déclin relatif: 7,8 %). Des déclins similaires ont été observés chez les hommes et les femmes et chez les individus âgés de plus ou de moins de 70 ans. L'entrée en vigueur de la législation n'a néanmoins pas été liée à une variation notable du taux d'AVC ischémique, de discopathie dégénérative et de cancer du côlon. Un brusque déclin de l'incidence d'infarctus du myocarde a également été observé lorsque des données sur les particules fines respirables ont été incluses dans une analyse portant sur Santiago.

**Conclusion:**

L'entrée en vigueur d'une législation antitabac stricte au Chili a été associée à un déclin brusque, quasi immédiat, de l'incidence d'infarctus du myocarde.

## Resumen

**Objetivo:**

Evaluar el impacto de una prohibición del tabaco completa en espacios cerrados sobre la incidencia de infartos de miocardio agudos en Chile.

**Métodos:**

El estudio basado en la población involucró a residentes de zonas urbanas, donde vive el 80% de la población chilena, de 20 años o más que han sufrido un infarto de miocardio. La incidencia de infartos de miocardio al mes y las tasas de mortalidad en centros sanitarios entre enero de 2011 y diciembre de 2014 derivaron de los centros de datos de admisión y mortalidad. Se utilizaron métodos de regresióndiscontinua para calcular el impacto casi inmediato en marzo de 2013 de la ley antitabaco sobre la incidencia de la enfermedad.Se realizó el mismo análisis para ictus isquémicos, discopatías degenerativas y cáncer de colon. Los datos sobre la concentración de partículas finas respirables se incluyeron en un análisis adicional de la incidencia de infartos de miocardio en la zona metropolitana de Santiago.

**Resultados:**

La entrada en vigor de la ley antitabaco se relacionó con una reducción abrupta y casi inmediata de 0,639 casos de infartos de miocardio por cada 100 000 adultos al mes (intervalo de confianza, IC, del 95%: del 0,242 al 1,036; descenso relativo: 7,8%). Se observaron reducciones similares en hombres y mujeres y en personas mayores y menores de 70 años. No obstante, la puesta en vigor de la ley no se relacionó con un cambio importante en la tasa de ictus isquémicos, discopatías degenerativas y cáncer de colon. También se observó una reducción abrupta de la incidencia de infartos de miocardio cuando se incluyeron los datos sobre partículas respirables en un análisis en Santiago.

**Conclusión:**

La entrada en vigor de una ley antitabaco exhaustiva en Chile se asoció con una reducción abrupta y casi inmediata de la incidencia de infartos de miocardio.

## ملخص

الغرض ****تقييم تأثير الحظر الكامل للتدخين في المناطق المغلقة على الإصابة بالذبحة القلبية الحادة (الاحتشاء الحاد لعضلة القلب) في شيلي.

الطريقة اشتملت الدراسة القائمة على الشرائح السكانية على سكان من المناطق الحضرية – والتي يقطن بها 80‏% من السكان في شيلي – تبدأ أعمارهم من سن 20 عامًا فما فوق ممن أُصيبوا بذبحة قلبية. وتم استخلاص النسبة الشهرية لمعدلات الإصابة بالذبحة القلبية في منشآت الرعاية الصحية في الفترة ما بين يناير/كانون الثاني 2011 وديسمبر/كانون الأول 2014 من قواعد بيانات الوفيات. وتمت الاستعانة بأساليب انقطاع التحوف (Regression discontinuity) للحد من التأثير شبه الفوري على وقوع الإصابة لتطبيق التشريع الخاص بمنع التدخين في مارس/آذار من عام 2013. وتم إجراء نفس التحليل على الذبحة الإقفارية، وداء القرص التنكسي، وسرطان القولون. كما تم تضمين البيانات الخاصة بتركيز الجسيمات الدقيقة القابلة للاستنشاق في تحليل إضافي للإصابة بالذبحة القلبية في منطقة سانتياغو الحضرية.

النتائج اقترن تطبيق التشريع الخاص بالحد من التدخين بحدوث انخفاض مفاجئ وشبه فوري إلى **0.639 ** حالة إصابة بالذبحة القلبية لكل 100 ألف بالغ في الشهر (بنسبة أرجحية مقدارها ‏95%: 0.242 إلى 1.036، بانخفاض نسبي بلغ: 7.8‏%). كما لوحظت نسب مشابهة للانخفاض بين الرجال والنساء في الفئات العمرية للأشخاص الذين تتخطى أعمارهم 70 وأقل، إلا أن تطبيق التشريع لم يقترن بحدوث تغيُّر ملموس في نسبة الإصابة بالسكتة الإقفارية، ولا داء القرص التنكسي، أو سرطان القولون. كما لوحظ أيضًا حدوث انخفاض مفاجئ في نسبة الإصابة بالسكتة القلبية عندما تم تضمين البيانات المتعلقة بالجسيمات الدقيقة القابلة للاستنشاق في تحليل شمل سانتياغو.

الاستنتاج ****اقترن تطبيق التشريع الموسع لمنع التدخين في شيلي بحدوث انخفاض فجائي وشبه فوري في حدوث الإصابة بالسكتة القلبية. ****

## 摘要

**目的:**

评估在封闭空间完全禁烟对智利急性心肌梗塞发病率的影响。

**方法:**

以人口为基础的研究涉及患有心肌梗塞的 20 岁或以上城市居民(智利 80% 的人口为城市居民)。 根据入院和死亡率数据库推导 2011 年 1 月至 2014 年 12 月期间医疗机构每月的心肌梗塞发病率和死亡率。 使用断点回归法估计 2013 年 3 月实施无烟立法对疾病发生率的即时影响。针对缺血性中风、退行性椎间盘疾病和结肠癌执行了同样的分析。 关于圣地亚哥大都会地区心肌梗塞发病率的补充分析中包含可吸入颗粒物浓度相关数据。

**结果:**

无烟立法实施后每月 100000 个成人中的心肌梗塞发病率突然立即减少 0.639 例(95% 置信区间,CI: 0.242 至 1.036;相对下降: 7.8%). 基于男性和女性以及 70 岁以上和 70 岁以下的人口进行分析时,发现了类似下降情况。 但是,立法实施后缺血性中风、退行性椎间盘疾病或结肠癌的发病率并无明显变化。 针对圣地亚哥的分析中包含可吸入颗粒物相关数据时,还发现心肌梗塞发病率突然下降。

**结论:**

智利广泛实施无烟立法促使心肌梗塞发病率突然立即下降。****

## Резюме

**Цель:**

Оценить влияние полного запрета на курение в закрытых помещениях на заболеваемость острым инфарктом миокарда в Чили.

**Методы:**

В популяционном исследовании участвовали жители городских районов, где проживает 80 % населения Чили, в возрасте 20 лет и старше, которые перенесли инфаркт миокарда. Данные о ежемесячной заболеваемости и смертности от инфаркта миокарда в медицинских учреждениях в период с января 2011 г. по декабрь 2014 г. были получены из баз данных о госпитализации и смертности. Для оценки непосредственного влияния применения законодательства, запрещающего курение, в марте 2013 г. на заболеваемость были использованы методы разрывной регрессии. Тот же анализ был выполнен для частоты случаев ишемического инсульта, дегенеративного заболевания межпозвоночных дисков и рака толстой кишки. Данные о концентрации мелких вдыхаемых частиц были включены в дополнительный анализ заболеваемости инфарктом миокарда в столичном регионе Сантьяго.

**Результаты:**

Применение законодательства, запрещающего курение, привело к резкому, почти мгновенному снижению на 0,639 случая инфаркта миокарда на 100 000 человек во взрослой популяции в месяц (95 % доверительный интервал, ДИ: от 0,242 до 1,036; относительное снижение: 7,8 %). Подобное снижение наблюдалось у мужчин и женщин, а также у людей в возрасте старше 70 лет. Однако применение законодательства не было связано со значительным изменением частоты случаев ишемического инсульта, дегенеративного заболевания межпозвоночных дисков или рака толстой кишки. Резкое снижение заболеваемости инфарктом миокарда наблюдалось также при включении в анализ для Сантьяго данных о мелких вдыхаемых частицах.

**Вывод:**

Применение обширного законодательства, запрещающего курение, в Чили привело к резкому, почти мгновенному снижению заболеваемости инфарктом миокарда.

## Introduction

After ratifying the World Health Organization’s Framework Convention on Tobacco Control in June 2005, Chile enacted its first round of tobacco control legislation in May 2006.^1^ The law completely prohibited smoking in public transport, in cinemas and in or near schools, but permitted designated smoking spaces in public facilities and workplaces with more than 10 employees.^2^

In February 2013, Chile enacted a second round of strengthened tobacco control legislation, which took effect on 1 March 2013.^3^ The new law extended the complete prohibition of smoking to all enclosed spaces accessible to the public, all enclosed commercial spaces and all open and closed sports arenas.^4^ Compliance with the new prohibitions on public smoking was almost immediate and virtually complete. Once the law took effect, inspections of bars, restaurants, educational establishments, health-care institutions and other public spaces increased four-fold. Violations were identified in only 1.7% of inspections (unpublished data, Ministerio de Salud de Chile, 2016). Despite the new tobacco control measures, the prevalence of tobacco use in Chile has remained one of the highest in the Americas.^5^ Among people aged 12 to 64 years, the proportion who reported smoking in the past month declined from 43.6% in 2002 to 34.0% in 2012, but rose to 39.7% in 2014.^6^

Extensive international literature supports the association between smoke-free legislation and a subsequent reduction in the incidence of myocardial infarction.^7^ Moreover, it has been estimated that exposure to second-hand tobacco smoke increases the risk of myocardial infarction in nonsmokers by approximately 30%.^8^ As with active smoking, some of the adverse effects of exposure to second-hand smoke occur almost immediately, including endothelial dysfunction, activation of platelet aggregation and lower heart rate variability.^9^ In addition, short-term exposure to tobacco smoke has been reported to reduce the coronary flow velocity reserve in healthy young adults^10^ and aortic distensibility in smokers and nonsmokers.^11^ Longer-term effects include an increase in oxidative stress, a decrease in high-density lipoprotein cholesterol levels, systemic inflammatory effects and an acceleration of atherosclerosis.^8^^,^^12^ At least some of the adverse effects of exposure to second-hand smoke are reversible.^13^

In Chile, ischaemic heart disease is the second largest cause of death after cerebrovascular accident in both sexes.^14^ We decided to study the potential impact of Chile’s extensive prohibition of smoking in urban public and private spaces in March 2013 on hospitalizations and deaths from acute myocardial infarction. Previous studies in South America – in Argentina and Uruguay – investigated only hospitalizations but, nevertheless, reported a reduction in the incidence of myocardial infarction after smoke-free legislation was implemented.^15^^,^^16^ Ours is the first population-based study of the effects of smoke-free legislation in the region.

In view of the short-term adverse effects of second-hand smoke exposure and the rapid and near-complete compliance with Chile’s March 2013 smoking prohibition, we employed an analytic strategy based on a regression discontinuity method to determine whether a discrete change in the myocardial infarction rate occurred almost immediately after the prohibition took effect. This strategy avoids many of the pitfalls of before-and-after study designs, particularly their limited capacity to take into account other confounding influences that could concurrently affect the myocardial infarction rate, such as changes in public policy and environmental factors.^17^ Because of evidence that smoke-free legislation may also reduce the incidence of cerebrovascular accidents and that coronary and ischaemic cerebrovascular diseases share some pathological mechanisms,^18^^,^^19^ we repeated the analysis for ischaemic stroke. In addition, following other studies that employed diagnostic controls,^19^ we repeated our analysis for degenerative disc diseases and colon cancer. Finally, to address the possible confounding influence of air pollution, we performed an additional analysis that included the concentration of fine respirable particulates in the Santiago metropolitan area, where continuous data were available.

## Methods

We conducted a population-based study of ST-elevation and non-ST elevation myocardial infarction in people aged 20 years or more in Chile who were treated before and after March 2013 at a public or private hospital or outpatient facility, including primary and urgent care centres. We excluded rural municipalities with a low population density and a small population. We employed the regression discontinuity method^20^^,^^21^ to identify the near-immediate impact of smoking prohibition on the rates of myocardial infarction, ischaemic stroke and two conditions unaffected by exposure to second-hand smoke.

We obtained data from two Chilean Ministry of Health databases on all individual hospital discharges and deaths, respectively, recorded in the country during the period 2011 to 2014.^22^^,^^23^ We identified all patients with a primary or secondary diagnosis of acute myocardial infarction (i.e. codes I21 and I22 in the 10th revision of the *International statistical classification of diseases and related health problems, ICD-10*), ischaemic stroke (i.e. codes I63, I64 and I67), degenerative disc disease (i.e. codes M50 and M51) and colon cancer (i.e. code C18).

Chile is divided into 346 administrative divisions called *comunas*. Our study covered 123 *comunas* with a 2012 population^24^ of at least 60 000 or a population density of at least 60 people per square kilometre.^25^ Together, these urban *comunas* were home to 80% of the Chilean population of 17.4 million in 2012. We determined the number of people in each urban *comuna* diagnosed with a myocardial infarction or one of the comparison conditions in each month between January 2011 and December 2014. In the two databases, the variable *comuna* referred to the individual’s residence. We obtained the total number of cases of myocardial infarction and of other conditions by summing the number of people with these conditions discharged from hospital alive, which was derived from the hospital discharge database, and the number of deaths occurring in health-care facilities, which was the greater of the two figures derived from the hospital discharge and mortality databases. For 76% of *comuna*–month combinations, mortality data on deaths from myocardial infarction at health-care facilities, which included primary care centres and hospital emergency departments, were more inclusive than discharge data on hospital inpatients whose death was attributed to myocardial infarction.

We included 37 833 patients who had a myocardial infarction between 2011 and 2014: 30 119 were discharged from hospital alive and 7714 died. We excluded 11 213 patients who died at home or at a site other than a health-care facility and whose deaths were attributed to, but could not reliably be confirmed as being due to, myocardial infarction. Among the patients included, 69% were male and 65% were aged between 20 and 69 years. Overall, 81% of all myocardial infarctions recorded in the country occurred among residents of urban *comunas*.

We calculated the rate of myocardial infarction and of other conditions in each month as the ratio of the number of cases to the number of residents aged 20 years or more in 2012, as derived from census data.^24^ For myocardial infarction, we consider these ratios to be approximate incidence rates. For the comparison conditions, we describe them simply as rates. We also analysed the rate of myocardial infarction stratified by sex and by age. We considered two age groups: 20 to 69 years and 70 years or more.

To take into account the possible confounding effect of air pollution, we obtained data on the daily concentration of particulate matter smaller than 2.5 micrometres (*PM_2.5_*) in air in the Santiago metropolitan area between 2011 and 2014, which were available from 11 monitoring stations.^26^ We incorporated the mean monthly *PM_2.5_* concentration into an analysis of the 52 *comunas* in the Santiago metropolitan area, which was home to approximately 40% of the Chilean population in 2012.^24^ The study was exempt from ethical approval because data were obtained from only publicly available databases that contained no personal identifiers.

### Statistical analysis

We used the regression discontinuity method^20^^,^^21^ to analyse trends in the monthly rate of myocardial infarction and of other diagnoses. To derive the regression discontinuity equation, let the index *t *represent the number of months after March 2013. Thus, *t* ranges from –26 for the month of January 2011 to +21 for December 2014, whereas March 2013 corresponds to* t *= 0.** Let *y_t_*denote the rate of myocardial infarction (or of another condition) in month* t*. Let *X_t_* be a binary variable equal to 0 if *t* < 0 and equal to 1 if *t* ≥ 0. Thus,* X_t_* switches from a value of 0 before the complete prohibition of smoking in enclosed public and private commercial spaces to a value of 1 once the prohibition comes into effect in March 2013. Let* Z_t_* denote a vector of other binary covariates corresponding to the calendar month of disease occurrence (i.e. February to December, with January as the reference category) and, in our restricted analysis of data from the Santiago metropolitan area, the monthly *PM_2.5_* concentration. Our regression discontinuity specification is:
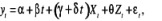
where α, β, γ, δ and θ are unknown parameters and ε*_t_* are independently identically distributed error terms with a zero mean.

The parameters of interest in Equation 1 are *γ* and *δ.* The parameter γ captures the time-independent, discrete change in the disease rate once the smoking prohibition has become effective. In geometric terms, it corresponds to the discontinuity, or gap, between preprohibition and postprohibition trend lines. By contrast, the parameter *δ* indicates the change in the temporal trend from before to after the prohibition has become effective. It corresponds to the difference between the slopes of the preprohibition and postprohibition trend lines. If smoke-free legislation resulted in a near-immediate reduction in the rate of myocardial infarction, we would expect the parameter *γ* to be negative and significantly different from zero. On the other hand, if the prohibition of smoking resulted in a gradual reduction in the rate of myocardial infarction over time, we would expect the parameter *δ* to be negative and significantly different from zero.

In graphical presentations of our results, we display the estimated trend lines derived from Equation 1 and the seasonally adjusted rates of myocardial infarction for each month from January 2011 to December 2014. To calculate seasonally adjusted myocardial infarction rates, we estimated the regression model,
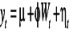
where *μ *and* ϕ *are unknown parameters, *W_t _*denotes the vector of binary covariates corresponding to the calendar month of disease occurrence (i.e. February to December, with January as the reference category) and *η_t_*is an error term. Using estimates, from this regression model, we then calculated seasonally adjusted myocardial infarction rates as *y_t_ -* *W_t_*. For the Santiago metropolitan area, we used the same method to compute the seasonally adjusted *PM_2.5_* concentration.

## Results

**Fig. 1:**
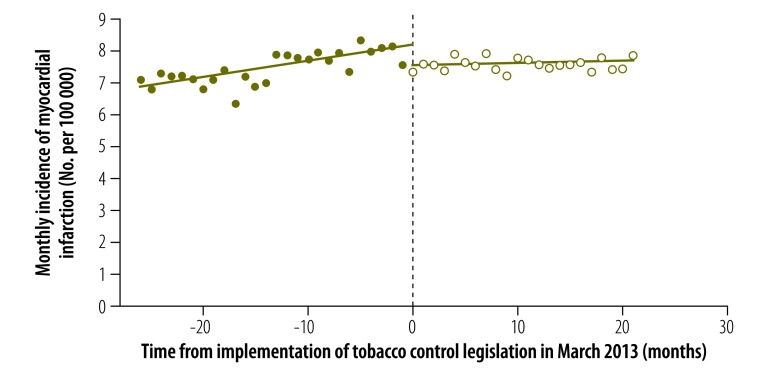
Incidence of myocardial infarction before and after enforcement of tobacco control legislation, Chile, 2011–2014

Notes: A second round of strengthened tobacco control legislation took effect on 1 March 2013. The data shown are for people aged 20 years and older living in urban areas of Chile. The circles show seasonally adjusted rates, as described in the text. Solid circles represent rates before March 2013 and open circles represent rates from March 2013 onward. The lines show the fitted values derived using the regression discontinuity model.

The results of our regression discontinuity analysis of the incidence of myocardial infarction are shown in Fig. 1 and Table 1. The estimated discontinuity in March 2013 at month 0 (corresponding to the parameter γ) and the change in temporal trend (corresponding to the parameter δ) were both statistically significant: = –0.639 (*P* = 0.002) and = –0.043 (*P* = 0.003). In relative terms, the near-immediate reduction in incidence was 7.8%, based upon the predicted incidence of 8.179 cases per 100 000 in March 2013 in the absence of smoke-free legislation. This reduction amounted to 64 fewer cases of myocardial infarction per month or 764 fewer cases per year in an adult population of 9.965 million in the urban *comunas* under study. Considering only data on hospital discharges did not significantly alter our findings: = –0.418 (*P* = 0.036) and = –0.049 (*P* = 0.001).

**Table 1:** Regression discontinuity analysis of the change in incidence of medical conditions with enforcement of tobacco control legislation,^a^ Chile, 2011–2014

Medical condition	Demographic group	Regression discontinuity model coefficients		
		Discrete change in disease rate ^b^		Change in temporal trend in disease rate ^c^
		Value (95% CI)		Value (95% CI)
Myocardial infarction	Both sexes, aged ≥ 20 years	–0.639 (–1.036 to –0.242)		–0.043 (–0.071 to –0.016)
Myocardial infarction	Males, aged ≥ 20 years	–0.778 (–1.462 to –0.095)		–0.069 (–0.117 to –0.022)
Myocardial infarction	Females, aged ≥ 20 years	–0.514 (–0.941 to –0.086)		–0.023 (–0.053 to 0.006)
Myocardial infarction	Both sexes, aged 20–69 years	–0.363 (–0.725 to –0.001)		–0.021 (–0.046 to 0.004)
Myocardial infarction	Both sexes, aged ≥ 70 years	–3.508 (–6.317 to –0.698)		–0.300 (–0.495 to –0.105)
Ischaemic stroke	Both sexes, aged ≥ 20 years	–0.188 (–1.073 to 0.697)		–0.084 (–0.145 to –0.022)
Degenerative disc disease	Both sexes, aged ≥ 20 years	0.124 (–0.422 to 0.669)		–0.019 (–0.057 to 0.019)
Colon cancer	Both sexes, aged ≥ 20 years	–0.112 (–0.421 to 0.197)		0.025 (0.004 to 0.047)
Myocardial infarction in the Santiago metropolitan area^d^	Both sexes, aged ≥ 20 years	–0.733 (–1.272 to –0.195)		–0.053 (–0.091 to –0.015)

CI: confidence interval.

^a^ A second round of strengthened tobacco control legislation took effect on 1 March 2013.

^b^ The parameter *γ* indicates the discrete change in disease rate associated with the enforcement of smoking legislation in March 2013.

^c^ The parameter *δ* indicates the change in the temporal trend of the disease rate associated with the enforcement of smoking legislation in March 2013.

^d^ An additional regression discontinuity model for the Santiago metropolitan area included the daily concentration of particulate matter in the air smaller than 2.5 micrometres (*PM_2.5_*).

**Fig. 2:**
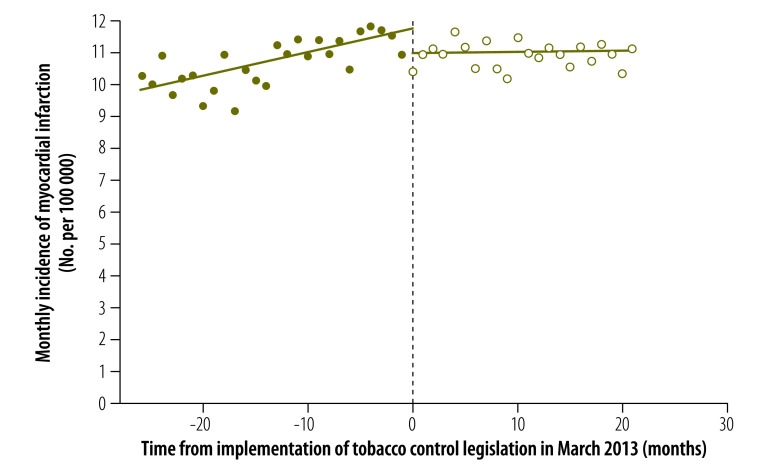
Incidence of myocardial infarction in men before and after enforcement of tobacco control legislation, Chile, 2011–2014

Notes: A second round of strengthened tobacco control legislation took effect on 1 March 2013. The data shown are for men aged 20 years and older living in urban areas of Chile. The circles show seasonally adjusted rates, as described in the text. Solid circles represent rates before March 2013 and open circles represent rates from March 2013 onward. The lines show the fitted values derived using the regression discontinuity model.

**Fig. 3:**
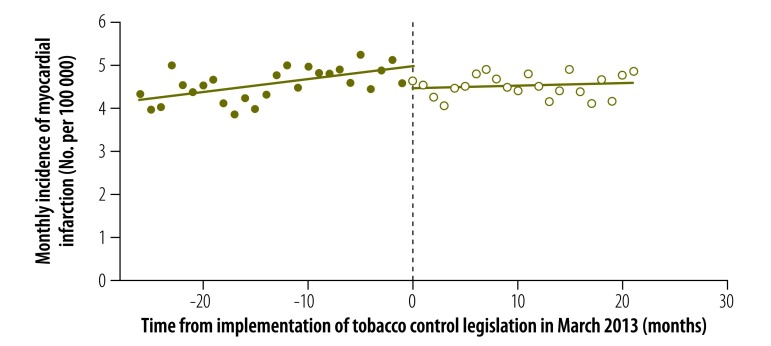
Incidence of myocardial infarction in women before and after enforcement of tobacco control legislation, Chile, 2011–2014

Notes: A second round of strengthened tobacco control legislation took effect on 1 March 2013. The data shown are for women aged 20 years and older living in urban areas of Chile. The circles show seasonally adjusted rates, as described in the text. Solid circles represent rates before March 2013 and open circles represent rates from March 2013 onward. The lines show the fitted values derived using the regression discontinuity model.

**Fig. 4:**
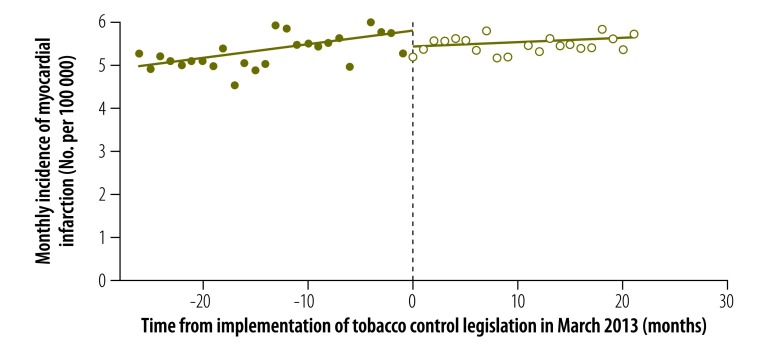
Incidence of myocardial infarction in people aged 20 to 69 years before and after enforcement of tobacco control legislation, Chile, 2011–2014

Notes: A second round of strengthened tobacco control legislation took effect on 1 March 2013. The data shown are for people aged 20 to 69 years living in urban areas of Chile. The circles show seasonally adjusted rates, as described in the text. Solid circles represent rates before March 2013 and open circles represent rates from March 2013 onward. The lines show the fitted values derived using the regression discontinuity model.

**Fig. 5:**
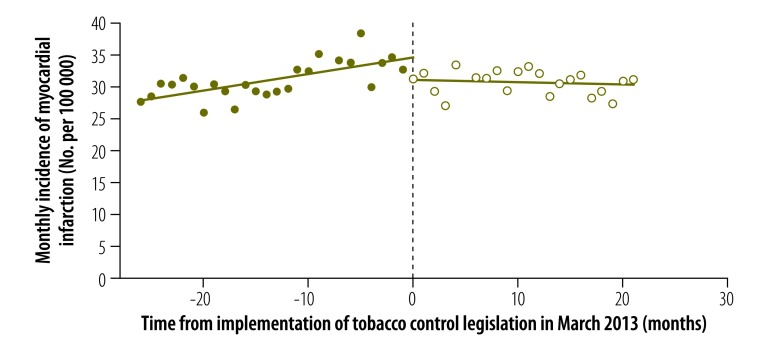
Incidence of myocardial infarction in people aged 70 years and older before and after enforcement of tobacco control legislation, Chile, 2011–2014

Notes: A second round of strengthened tobacco control legislation took effect on 1 March 2013. The data shown are for people aged 70 years and older living in urban areas of Chile. The circles show seasonally adjusted rates, as described in the text. Solid circles represent rates before March 2013 and open circles represent rates from March 2013 onward. The lines show the fitted values derived using the regression discontinuity model.

The results of our analysis of the change in the incidence of myocardial infarction stratified by sex are displayed in Fig. 2, Fig. 3 and Table 1. In males, both the estimated discontinuity and the change in temporal trend were statistically significant: = –0.778 (*P* = 0.027) and = –0.069 (*P* = 0.006). In females, the estimated discontinuity was statistically significant ( = –0.514, *P* = 0.020) but the estimated change in temporal trend was not significantly different from zero (= –0.023, *P* = 0.121). The results of the corresponding analysis stratified by age group are shown in Fig. 4, Fig. 5 and Table 1. The estimated discontinuities were statistically significant for both groups: *P* = 0.049 for individuals aged 20 to 69 years and *P* = 0.016 for those aged 70 years and older. In contrast, the estimated change in temporal trend was significant only for those aged 70 years and older: = –0.300 (*P* = 0.004).

**Fig. 6:**
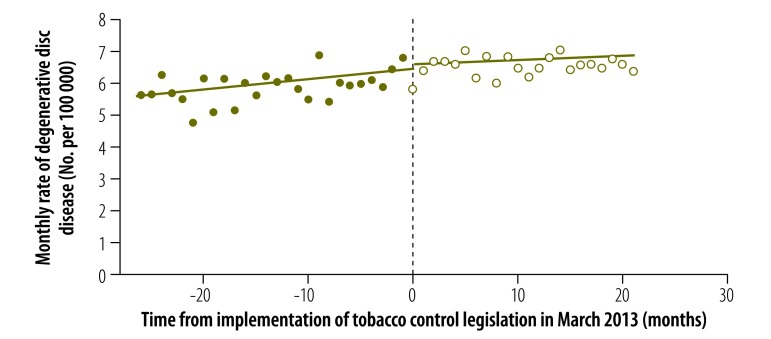
Rate of degenerative disc disease before and after enforcement of tobacco control legislation, Chile, 2011–2014

Notes: A second round of strengthened tobacco control legislation took effect on 1 March 2013. The data shown are for people aged 20 years and older living in urban areas of Chile. The circles show seasonally adjusted rates, as described in the text. Solid circles represent rates before March 2013 and open circles represent rates from March 2013 onward. The lines show the fitted values derived using the regression discontinuity model.

Our corresponding results for other conditions are also shown in Table 1. For ischaemic stroke, the estimated discontinuity was not significant ( = –0.188, *P* = 0.668), whereas the estimated change in temporal trend was negative and significantly different from zero ( = –0.084, *P* = 0.009). Expansion of the definition of stroke to include nontraumatic, intracerebral haemorrhage (i.e. *ICD-10* code I61) did not substantially alter our findings ( = –0.218, *P* = 0.650; and = –0.094, *P* = 0.008). Our findings for degenerative disc disease are shown in Fig. 6. Neither the estimated discontinuity nor the change in trend was statistically significant ( = 0.124, *P* = 0.648; and = –0.019, *P* = 0.306, respectively). For colon cancer, the estimated discontinuity was not significant ( = –0.112, *P* = 0.467), whereas the estimated change in trend was positive and significantly different from zero ( = 0.025, *P* = 0.023).

**Fig. 7:**
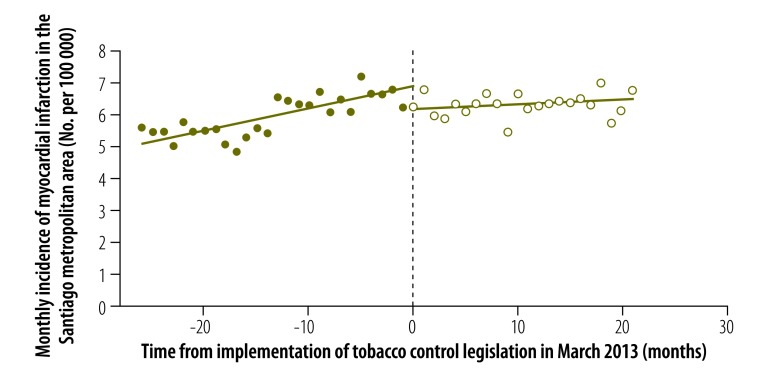
Incidence of myocardial infarction in the Santiago metropolitan area before and after enforcement of tobacco control legislation, Chile, 2011–2014

Notes: A second round of strengthened tobacco control legislation took effect on 1 March 2013. The data shown are for people aged 20 years and older living in the Santiago metropolitan area. The circles show seasonally adjusted rates, as described in the text. Solid circles represent rates before March 2013 and open circles represent rates from March 2013 onward. The lines show the fitted values derived using the regression discontinuity model.

**Fig. 8:**
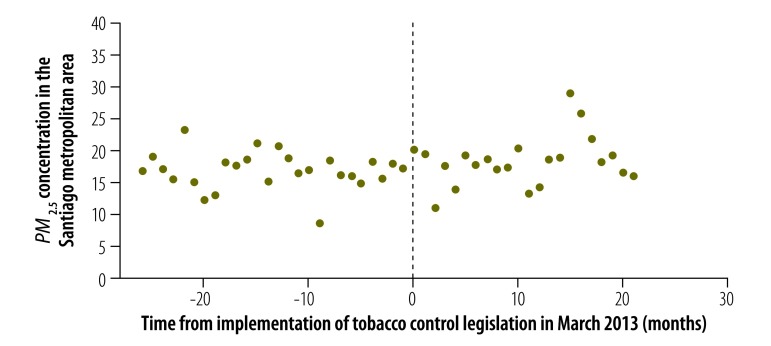
Atmospheric *PM_2.5_* concentration in the Santiago metropolitan area before and after enforcement of tobacco control legislation, Chile, 2011–2014

The estimated seasonally adjusted incidence of myocardial infarction in the Santiago metropolitan area is shown in Fig. 7 and the corresponding seasonally adjusted *PM_2.5_* concentration is shown in Fig. 8. In a regression discontinuity model that included the *PM_2.5_* concentration, both the estimated discontinuity in the incidence of myocardial infarction in March 2013 ( = –0.733, *P* = 0.009) and the estimated change in trend ( = –0.053, *P* = 0.008) remained significant (Table 1). The coefficient of the *PM_2.5_* term in the regression model was not significantly different from zero (*P* = 0.771).

*PM_2.5_*: particulate matter smaller than 2.5 micrometres.

Notes: A second round of strengthened tobacco control legislation took effect on 1 March 2013. Particulate matter concentrations were available from 11 monitoring stations in Santiago.

## Discussion

We observed a significant abrupt reduction in the incidence of myocardial infarction in urban municipalities in Chile in the same month that smoke-free legislation went into effect. This abrupt decline was observed in both sexes and in people aged 20 to 69 years and 70 years and older. There was no corresponding significant abrupt reduction in the rate of ischaemic stroke, degenerative disc disease or colon cancer. In an analysis restricted to the Santiago metropolitan area, inclusion of the concentration of fine respirable particulates had no detectable effect on our regression estimates.

Many prior studies have compared trends in the incidence of myocardial infarction during extended periods before and after the enforcement of public smoking bans. Our study follows the lead of a few others in assessing the near-immediate effect of a public smoking ban, as captured by the discontinuity in the estimated trend lines.^16^^,^^19^^,^^27^^–^^29^ The rationale for this approach is based on evidence that exposure to second-hand smoke has a near-immediate adverse effect on the risk of myocardial infarction and that enforcement of, and compliance with, smoke-free legislation is also near-immediate.

We also observed that the slope of the temporal trend line of myocardial infarction incidence was significantly smaller in the 20-month period after Chile’s smoke-free legislation went into effect than it was before. Although smoke-free legislation may have contributed to these longer-term trends, other factors, such as the additional tobacco control measures enacted as part of the 2013 law, could have been responsible.^3^ For example, the real price of cigarettes increased by approximately 30% between 2011 and 2014.^30^ In addition, the increased use of troponin testing in Chilean hospitals to diagnose non-ST elevation myocardial infarction could also have influenced disease rates. Although the prevalence of all cardiovascular risk factors except diabetes remained stable between 2003 and 2010,^31^ no comparable data were available for the period 2011 to 2014. These potentially confounding factors could have influenced trends in disease incidence in the 2 years after the enforcement of smoke-free legislation, but they are less likely to have contributed to the abrupt decline observed in the month immediately after the legislation went into effect.

Inclusion of the *PM_2.5_* concentration did not alter our finding of a significant abrupt decline in the incidence of myocardial infarction after March 2013. Nor did the temporal pattern of air pollution show any relation to incidence. In June 2011, before the enforcement of smoke-free legislation, the Puyehue–Cordón Caulle volcanic complex erupted in southern Chile. The resulting emission of particulates and toxic gases could have affected disease incidence. However, the dominant wind direction during the eruption was from west to east, which dispersed ash clouds into Argentina, Brazil and Uruguay.^32^

We estimated that the relative decline in the incidence of myocardial infarction in the urban municipalities included in our analysis was 7.8%. This figure is towards the lower end of the 4 to 52% range reported in meta-analyses.^33^^,^^34^ However, our study focused solely on the near-immediate decline in incidence, whereas most other studies took into account longer-term effects.

Several studies of the impact of smoke-free legislation employed geographical controls.^15^^,^^19^^,^^29^^,^^35^^–^^40^ We could not use this study design because Chile’s 2013 law was adopted on a nationwide basis. Here, we hypothesized that people living in urban areas with a higher population or population density would have greater exposure to second-hand smoke and would thus experience a marked reduction in the likelihood of a myocardial infarction after the enforcement of smoke-free legislation. This hypothesis was reinforced by the substantially higher smoking prevalence in urban areas of Chile,^41^ as well as by the observed dose–response association between the extent of second-hand smoke exposure and the risk of myocardial infarction.^42^^,^^43^ Following other studies,^19^ we employed diagnostic controls.

Although there is some evidence that smoke-free legislation can reduce the incidence of ischaemic cerebrovascular accidents,^18^^,^^19^ we detected a significant decline only in the slope of the trend line with the enforcement of legislation in Chile – there was no abrupt reduction in the rate of strokes. Our analysis may have been limited by the misclassification of cerebrovascular events. Among the three *ICD-10* categories we employed, I64 (i.e. stroke, not specified as haemorrhage or infarction) and I67 (i.e. other cerebrovascular diseases) accounted for about 30 and 45% of events, respectively. Inclusion of I61 (i.e. nontraumatic, intracerebral haemorrhage) did not alter our findings substantially.

Most studies of the impact of smoke-free legislation on the incidence of myocardial infarction have relied solely upon data on hospitalizations,^15^^–^^17^^,^^19^^,^^27^^–^^29^^,^^35^^–^^37^^,^^40^^,^^44^^–^^47^ although some have analysed mortality data, either alone or with hospitalization data.^38^^,^^39^^,^^48^ Here, we employed a population-based approach, combining both hospitalization and mortality data. Still, we included only 37 833 cases of myocardial infarction diagnosed in a hospital or other health-care facility. Out of concern for diagnostic reliability, we excluded 11 213 deaths outside of health-care facilities that were attributed to, but not confirmed as, myocardial infarction.

Our study has two additional limitations. First, we lacked information on each individual’s smoking status and thus could not directly verify that the decrease in myocardial infarctions among nonsmokers resulted from reduced exposure to second-hand smoke. Second, our follow-up period ended in 2014. Longer follow-up may have improved the precision of our estimates of the post-ban trend line and of the immediate reduction in the incidence of myocardial infarction.

Our findings support the view that the smoke-free legislation enacted in 2013 in Chile had a significant favourable effect on the health of the population. These results will be useful in evaluating national policies for tobacco control and myocardial infarction prevention.

## References

[1] Gobierno de Chile. Ley no. 20.105: modifica la ley no. 19.419, en Materias Relativas a La Publicidad y El Consumo Del Tabaco. Santiago: Biblioteca del Congreso Nacional de Chile; 2006 (in Spanish).

[2] Valenzuela Schmidt MT. Chile: Situación del tabaquismo a cinco años de la ratificación del Convenio Marco para el Control del Tabaco y los desafíos pendientes. Informe de Chile Libre de Tabaco. El Bosque: Fundación Educación Popular en Salud; 2010. Available from: http://www.chilelibredetabaco.cl/descargas/Informe_Chile_5_anos_CMCT_EPES_2010.pdf [cited 2017 Aug 2] (in Spanish).

[3] Gobierno de Chile. Ley no. 20.660: modifica ley no. 19.419, en Materia de Ambientes Libres de Humo de Tabaco. Santiago: Biblioteca del Congreso Nacional de Chile; 2013 (in Spanish).

[4] Ministerio de Salud de Chile. Ley 20.660, que modifica la ley no. 19.419 en Materia de Ambientes Libres de Humo del Tabaco. Geneva: World Health Organization; 2013. Available from: http://www.who.int/fctc/implementation/news/130201_Ley_20.660_de_Tabaco.pdf?ua=1 [cited 2016 Nov 14] (in Spanish).

[5] Informe sobre control de tabaco en la región de las Américas: a 10 años del Convenio Marco de la Organización Mundial de Salud para el Control de Tabaco. Washington DC: Organización Panamericana de la Salud; 2016. Available from: http://iris.paho.org/xmlui/bitstream/handle/123456789/28380/9789275318867_spa.pdf?sequence=1&isAllowed=y&ua=1 [cited 2017 Aug 2] (in Spanish).

[6] Observatorio Chileno de Drogas. Décimo primer estudio nacional de drogas en población general de Chile, 2014. Santiago: Servicio Nacional para la Prevención y Rehabilitación del Consumo de Drogas y Alcohol, SENDA; 2015. Available from: http://www.senda.gob.cl/media/estudios/PG/2014_EstudioDrogas_Poblacion_General.pdf [cited 2017 Aug 2] (in Spanish).

[7] Frazer K, Callinan JE, McHugh J, van Baarsel S, Clarke A, Doherty K, et al. Legislative smoking bans for reducing harms from secondhand smoke exposure, smoking prevalence and tobacco consumption. Cochrane Database Syst Rev. 2016 2 4;2:CD005992. 10.1002/14651858.CD005992.pub3 26842828 PMC6486282

[8] Barnoya J, Glantz SA. Cardiovascular effects of secondhand smoke: nearly as large as smoking. Circulation. 2005 5 24;111(20):2684–98. 10.1161/CIRCULATIONAHA.104.492215 15911719

[9] Felber Dietrich D, Schwartz J, Schindler C, Gaspoz JM, Barthélémy JC, Tschopp JM, et al.; SAPALDIA-team. Effects of passive smoking on heart rate variability, heart rate and blood pressure: an observational study. Int J Epidemiol. 2007 8;36(4):834–40. 10.1093/ije/dym031 17440032

[10] Otsuka R, Watanabe H, Hirata K, Tokai K, Muro T, Yoshiyama M, et al. Acute effects of passive smoking on the coronary circulation in healthy young adults. JAMA. 2001 7 25;286(4):436–41. 10.1001/jama.286.4.436 11466122

[11] Stefanadis C, Vlachopoulos C, Tsiamis E, Diamantopoulos L, Toutouzas K, Giatrakos N, et al. Unfavorable effects of passive smoking on aortic function in men. Ann Intern Med. 1998 3 15;128(6):426–34. 10.7326/0003-4819-128-6-199803150-00002 9499325

[12] Glantz SA, Parmley WW. Passive smoking and heart disease. Epidemiology, physiology, and biochemistry. Circulation. 1991 1;83(1):1–12. 10.1161/01.CIR.83.1.1 1984876

[13] Raitakari OT, Adams MR, McCredie RJ, Griffiths KA, Celermajer DS. Arterial endothelial dysfunction related to passive smoking is potentially reversible in healthy young adults. Ann Intern Med. 1999 4 6;130(7):578–81. 10.7326/0003-4819-130-7-199904060-00017 10189327

[14] Defunciones y mortalidad por causas. Santiago: Departamento de Estadísticas e Información de Salud; 2016.Available from: http://www.deis.cl/defunciones-y-mortalidad-por-causas/ [cited 2016 Nov 7] (in Spanish).

[15] Ferrante D, Linetzky B, Virgolini M, Schoj V, Apelberg B. Reduction in hospital admissions for acute coronary syndrome after the successful implementation of 100% smoke-free legislation in Argentina: a comparison with partial smoking restrictions. Tob Control. 2012 7;21(4):402–6. 10.1136/tc.2010.042325 21602536

[16] Sebrié EM, Sandoya E, Bianco E, Hyland A, Cummings KM, Glantz SA. Hospital admissions for acute myocardial infarction before and after implementation of a comprehensive smoke-free policy in Uruguay: experience through 2010. Tob Control. 2014 11;23(6):471–2. 10.1136/tobaccocontrol-2012-050954 25324157 PMC4358818

[17] Galán I, Simón L, Flores V, Ortiz C, Fernández-Cuenca R, Linares C, et al. Assessing the effects of the Spanish partial smoking ban on cardiovascular and respiratory diseases: methodological issues. BMJ Open. 2015 12 1;5(12):e008892. 10.1136/bmjopen-2015-008892 26628524 PMC4679921

[18] Chapter 8. Cardiovascular diseases. In: U.S. Department of Health and Human Services. The health consequences of smoking—50 years of progress: a report of the Surgeon General. Rockville: U.S. Department of Health and Human Services, Centers for Disease Control and Prevention, National Center for Chronic Disease Prevention and Health Promotion, Office on Smoking and Health; 2014. pp. 411–58. Available from: https://www.surgeongeneral.gov/library/reports/50-years-of-progress [cited 2017 Aug 2].

[19] Naiman A, Glazier RH, Moineddin R. Association of anti-smoking legislation with rates of hospital admission for cardiovascular and respiratory conditions. CMAJ. 2010 5 18;182(8):761–7. 10.1503/cmaj.091130 20385737 PMC2871198

[20] Thistlethwaite DL, Campbell DT. Regression-discontinuity analysis: an alternative to the ex post facto experiment. J Educ Psychol. 1960;51(6):309–17. 10.1037/h0044319

[21] Imbens GW, Lemieux T. Regression discontinuity designs: a guide to practice. J Econom. 2008;142(2):615–35. 10.1016/j.jeconom.2007.05.001

[22] Bases de datos egresos hospitalarios. Santiago: Departamento de Estadísticas e Información de Salud; 2016. Available from: http://www.deis.cl/bases-de-datos-egresos-hospitalarios/ [cited 2016 Sep 20] (in Spanish).

[23] Bases de datos defunciones. Santiago: Departamento de Estadísticas e Información de Salud; 2016. Available from: http://www.deis.cl/bases-de-datos-defunciones/ [cited 2016 Oct 5] (in Spanish).

[24] Comunas: población estimada al 30 de junio por sexo y edad simple 2002–2020. Base de datos (XLS, 13 MB). Demográficas y vitales: productos estadísticos. Santiago: Instituto Nacional de Estadísticas Chile; 2016. Available from: http://www.ine.cl/canales/chile_estadistico/familias/demograficas_vitales.php [cited 2016 Sep 25] (in Spanish).

[25] División político administrativa y censal 2007. Santiago: Instituto Nacional de Estadísticas; 2008. Available from: http://historico.ine.cl/canales/chile_estadistico/territorio/division_politico_administrativa/pdf/DPA_COMPLETA.pdf [cited 2016 Oct 22] (in Spanish).

[26] Sistema de Información Nacional de Calidad del Aire (SINCA). Región metropolitana de Santiago: Estaciones de monitoreo de la calidad del aire. Santiago: Ministerio del Medio Ambiente; 2017. Available from: http://sinca.mma.gob.cl/index.php/region/index/id/M [cited 2017 Jun 26]. (in Spanish).

[27] Barone-Adesi F, Gasparrini A, Vizzini L, Merletti F, Richiardi L. Effects of Italian smoking regulation on rates of hospital admission for acute coronary events: a country-wide study. PLoS One. 2011 3 2;6(3):e17419. 10.1371/journal.pone.0017419 21399685 PMC3047543

[28] Juster HR, Loomis BR, Hinman TM, Farrelly MC, Hyland A, Bauer UE, et al. Declines in hospital admissions for acute myocardial infarction in New York state after implementation of a comprehensive smoking ban. Am J Public Health. 2007 11;97(11):2035–9. 10.2105/AJPH.2006.099994 17901438 PMC2040364

[29] Bartecchi C, Alsever RN, Nevin-Woods C, Thomas WM, Estacio RO, Bartelson BB, et al. Reduction in the incidence of acute myocardial infarction associated with a citywide smoking ordinance. Circulation. 2006 10 3;114(14):1490–6. 10.1161/CIRCULATIONAHA.106.615245 17000911

[30] Triunfo P, Harris J, Balsa A. [Evaluation of Uruguay’s antismoking campaign: progress and challenges at ten years]. Rev Panam Salud Publica. 2016 10;40(4):256–62. 28001202

[31] Encuesta Nacional de Salud ENS 2009–2010 [slide presentation]. Santiago: Ministerio de Salud, Gobierno de Chile; 2011. Available from: http://www.dinta.cl/wp-dintacl/wp-content/uploads/Presentacion-ENSalud-2010.pdf [cited 2017 Jun 26] (in Spanish).

[32] Lagos F, Martinez P, Morales C. Erupciones recientes del volcán Chaitén y Cordón Caulle. Santiago: Departamento de Geología, Universidad de Chile; 2016. Available from: http://www.geologia.uchile.cl/erupciones-recientes-del-volcan-chaiten-y-cordon-caulle [cited 2017 Jun 12] (in Spanish).

[33] Lightwood JM, Glantz SA. Declines in acute myocardial infarction after smoke-free laws and individual risk attributable to secondhand smoke. Circulation. 2009 10 6;120(14):1373–9. 10.1161/CIRCULATIONAHA.109.870691 19770392 PMC2967202

[34] Lin H, Wang H, Wu W, Lang L, Wang Q, Tian L. The effects of smoke-free legislation on acute myocardial infarction: a systematic review and meta-analysis. BMC Public Health. 2013 5 31;13(1):529. 10.1186/1471-2458-13-529 23721370 PMC3671962

[35] Di Valentino M, Muzzarelli S, Limoni C, Porretta AP, Rigoli A, Barazzoni F, et al. Reduction of ST-elevation myocardial infarction in Canton Ticino (Switzerland) after smoking bans in enclosed public places–No Smoke Pub Study. Eur J Public Health. 2015 4;25(2):195–9. 10.1093/eurpub/cku067 24895081

[36] Bruintjes G, Bartelson BB, Hurst P, Levinson AH, Hokanson JE, Krantz MJ. Reduction in acute myocardial infarction hospitalization after implementation of a smoking ordinance. Am J Med. 2011 7;124(7):647–54. 10.1016/j.amjmed.2011.02.022 21683831

[37] Barr CD, Diez DM, Wang Y, Dominici F, Samet JM. Comprehensive smoking bans and acute myocardial infarction among Medicare enrollees in 387 US counties: 1999-2008. Am J Epidemiol. 2012 10 1;176(7):642–8. 10.1093/aje/kws267 22986145 PMC3530376

[38] Rodu B, Peiper N, Cole P. Acute myocardial infarction mortality before and after state-wide smoking bans. J Community Health. 2012 4;37(2):468–72. 10.1007/s10900-011-9464-5 21877107

[39] Dove MS, Dockery DW, Mittleman MA, Schwartz J, Sullivan EM, Keithly L, et al. The impact of Massachusetts’ smoke-free workplace laws on acute myocardial infarction deaths. Am J Public Health. 2010 11;100(11):2206–12. 10.2105/AJPH.2009.189662 20864706 PMC2951939

[40] Sargent RP, Shepard RM, Glantz SA. Reduced incidence of admissions for myocardial infarction associated with public smoking ban: before and after study. BMJ. 2004 4 24;328(7446):977–80. 10.1136/bmj.38055.715683.55 15066887 PMC404491

[41] 5.5. Prevalencia de fumador actual según ruralidad y sexo. In: Encuesta Nacional de Salud ENS Chile 2009–2010. Tomo II. V. Resultados Santiago. p. 155. Santiago: Ministerio de Salud, Gobierno de Chile; 2012 (in Spanish).

[42] He J, Vupputuri S, Allen K, Prerost MR, Hughes J, Whelton PK. Passive smoking and the risk of coronary heart disease–a meta-analysis of epidemiologic studies. N Engl J Med. 1999 3 25;340(12):920–6. 10.1056/NEJM199903253401204 10089185

[43] The health consequences of involuntary exposure to tobacco smoke: a report of the Surgeon General. Atlanta: Centers for Disease Control and Prevention; 2006. Available from: https://www.ncbi.nlm.nih.gov/books/NBK44324/ [cited 2017 Aug 2]. 20669524

[44] Jan C, Lee M, Roa R, Herrera V, Politis M, Motta J. The association of tobacco control policies and the risk of acute myocardial infarction using hospital admissions data. PLoS One. 2014 2 10;9(2):e88784. 10.1371/journal.pone.0088784 24520421 PMC3919809

[45] Pell JP, Haw S, Cobbe S, Newby DE, Pell AC, Fischbacher C, et al. Smoke-free legislation and hospitalizations for acute coronary syndrome. N Engl J Med. 2008 7 31;359(5):482–91. 10.1056/NEJMsa0706740 18669427

[46] Hahn EJ, Rayens MK, Burkhart PV, Moser DK. Smoke-free laws, gender, and reduction in hospitalizations for acute myocardial infarction. Public Health Rep. 2011 Nov-Dec;126(6):826–33. 22043098 10.1177/003335491112600608 PMC3185318

[47] Liu A, Guzman Castillo M, Capewell S, Lucy J, O’Flaherty M. Reduction in myocardial infarction admissions in Liverpool after the smoking ban: potential socioeconomic implications for policymaking. BMJ Open. 2013 11 25;3(11):e003307. 10.1136/bmjopen-2013-003307 24282240 PMC3845049

[48] Agüero F, Dégano IR, Subirana I, Grau M, Zamora A, Sala J, et al. Impact of a partial smoke-free legislation on myocardial infarction incidence, mortality and case-fatality in a population-based registry: the REGICOR Study. PLoS One. 2013;8(1):e53722. 10.1371/journal.pone.0053722 23372663 PMC3553094

