# Corrigenda

**DOI:** 10.2471/BLT.17.101117

**Published:** 2017-11-01

In: Nazzal C, Harris JE. Lower incidence of myocardial infarction after smoke-free legislation enforcement in Chile. Bull World Health Organ. 2017 October 1;95(10):674–682. http://dx.doi.org/10.2471/BLT.16.189894:

• on page 674, the last six words in the last line of the second paragraph should read “but rose to 34.7% in 2014.”;

• on page 676, middle column, eight line, the delta variable should have a circumflex accent;

• on page 676, Table 1, the subtitle in the third column should have the Greek letter gamma in parentheses before the superscript b and the subtitle in the fourth column should have the Greek letter delta in parentheses before the superscript c;

• on page 677, first column, twelfth line of the last paragraph the Greek letter gamma with a circumflex should be replaced with the Greek letter delta with a circumflex. 

